# An Inverse Relationship Links Temperature and Substrate Apparent Affinity in the Ion-Coupled Cotransporters rGAT1 and KAAT1

**DOI:** 10.3390/ijms131215565

**Published:** 2012-11-22

**Authors:** Antonio Peres, Alessandra Vollero, Eleonora Margheritis, Francesca D’Antoni, Elena Bossi

**Affiliations:** Department of Biotechnology and Life Sciences, University of Insubria, Via Dunant, 3, 21100 Varese, Italy; E-Mails: alessandra.vollero@uninsubria.it (A.V.); eleonora.margheritis@uninsubria.it (E.M.); francesca.dantoni@uninsubria.it (F.D.); elena.bossi@uninsubria.it (E.B.)

**Keywords:** transporter, temperature, affinity, rGAT1, KAAT1, electrophysiology

## Abstract

The effects of temperature on the operation of two ion-coupled cotransporters of the SLC6A family, namely rat GAT1 (SLC6A1) and KAAT1 (SLC6A19) from *Manduca sexta*, have been studied by electrophysiological means in *Xenopus laevis* oocytes expressing these proteins. The maximal transport-associated current (*I*_max_) and the apparent substrate affinity (*K*_05_) were measured. In addition to the expected increase in transport rate (*Q*_10_ = 3–6), both transporters showed greater *K*_05_ values (*i.e.*, a decrease in apparent affinity) at higher temperatures. The transport efficiency, estimated as *I*_max_/*K*_05_, increased at negative potentials in both transporters, but did not show statistically significant differences with temperature. The observation that the apparent substrate affinity is inversely related to the transport rate suggests a kinetic regulation of this parameter. Furthermore, the present results indicate that the affinities estimated at room temperature for mammalian cotransporters may not be simply extrapolated to their physiological operating conditions.

## 1. Introduction

Functional studies of ion-coupled cotransporters have strongly taken advantage of the possibility of heterologous expression in *Xenopus* oocytes, which may be studied with relatively simple electrophysiological techniques, such as the two-electrode voltage-clamp (TEVC). This approach suffers, however, of an important drawback when mammalian transporters are investigated, because temperatures above 30 °C are not easily tolerated by the oocytes, and therefore, most studies have been performed at room temperature (20 to 25 °C) [1–4].

In the relatively few papers reporting the effects of temperature on the activity of cotransporters [5–9], the focus of the observations was generally on the rate of transport and on the effects on the presteady-state currents, *i.e.*, the electrical signal arising from the initial steps of the transport cycle [1,10,11]. In a recent work from our laboratory on the rabbit intestinal oligopeptide transporter PepT1 [12], we observed that the apparent substrate affinity was significantly affected by temperature. In particular, a decrease in apparent affinity was measured in the physiological voltage range when increasing the temperature from 20 to 30 °C. This effect was associated with a strong increase in the maximal transport current and with an acceleration of the kinetics of the presteady-state currents. All together, these observations point to a kinetic interpretation of the apparent substrate affinity in which a greater substrate concentration is required to keep up with the ability of a faster turnover of the transporter. In fact, we have already reported this kind of relationship in the GABA transporter GAT1 [13–16].

To better investigate these points, we have studied the effects of temperature on the apparent substrate affinity in two other transporters, namely the rat neuronal GABA transporter GAT1 and the neutral amino acid transporter KAAT1, cloned from the gut of the invertebrate *Manduca sexta*[17]. The aim of the investigation was first of all to verify if the apparent substrate affinity was similarly affected by temperature and, secondarily, to examine the overall efficiency of the transport, considering in addition that, while rGAT1 (and PepT1) are from mammalian (homeotherm) animals, KAAT1 originates from a poikilotherm invertebrate [18].

## 2. Results and Discussion

### 2.1. Apparent Affinity Changes Induced by Temperature in rGAT1

**Figure 1:**
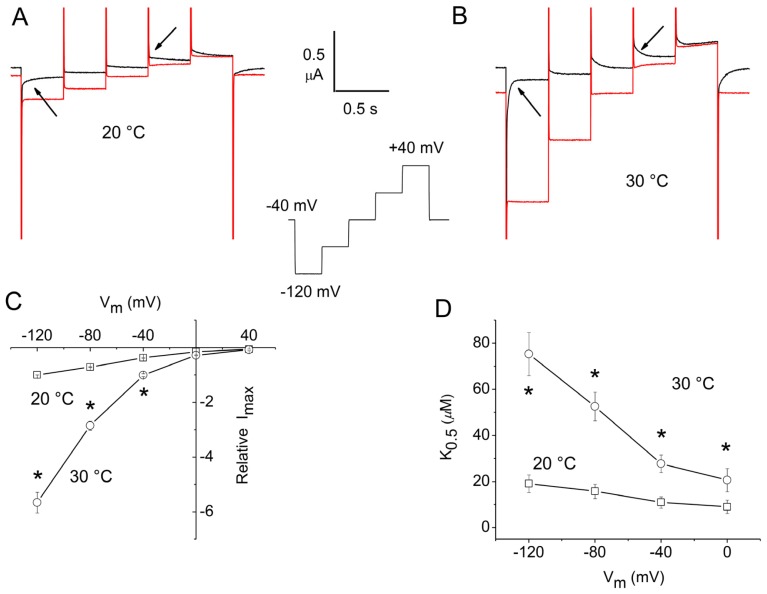
Temperature effects on the kinetic properties of rGAT1. Top row: currents in the absence (black) or presence (red) of 300 μM GABA at 20 °C (**A**) and 30 °C (**B**), in response to the staircase voltage protocol shown in the inset. The arrows point to the presteady-state currents that disappear in the presence of GABA. The bottom row shows the results of the Michaelis-Menten analysis performed on dose-response curves obtained using the same experimental protocol: voltage dependence of *I*_max_ (**C**) and of *K*_05_ (**D**) at the indicated temperatures. Data are means ± SE from seven oocytes (three batches). The current data were normalized to the value at −120 mV and 20 °C for each oocyte before averaging. The *K*_05_ value at +40 mV is omitted because its estimate is unreliable. Data marked with asterisks were significantly different at the two temperatures (Student’s *t*-test, *p* < 0.05).

For these experiments, a staircase voltage protocol was used to minimize temperature fluctuations during the recording [12]. For rGAT1, the protocol was applied from a holding potential (*V*_h_) = −40 mV and consisted of five steps spanning the range from −120 to +40 mV in 40 mV intervals. The steps were 400 ms in duration to allow for the complete decline of the presteady-state currents, particularly at the lower temperature. The current traces in response to this protocol at 20 and 30 °C are shown in Figure 1A,B, in the absence or presence of GABA 300 μM. As expected [4,19,20], the presteady-state currents visible in the absence of GABA (arrows) are abolished by its addition. It may be noted that these currents are strongly accelerated at the higher temperature, and accordingly, the transport-associated currents become significantly larger. Dose-response experiments were performed in a series of oocytes at these two temperatures in order to obtain the values of the maximal transport current (*I*_max_) and of the substrate concentration, eliciting the half-maximal current (*K*_05_) by fitting the data to the Michaelis-Menten equation:

(1) $$I=\frac{I_{\text{max}}}{1+\frac{K_{05}}{\left[\text{substrate}\right]}}$$

Figure 1C,D show the results of this analysis: *I*_max_ is strongly increased at 30 °C compared to 20 °C; the *Q*_10_ of the effect is variable with voltage from 2.7 at −40 mV up to almost 6 at −120 mV. This value is higher than those reported before [6,7], however, it must be noted that the previous data were obtained using a fixed GABA concentration and therefore, did not account for changes in apparent affinity. Indeed, the effect of temperature on *K*_05_ may be seen in Figure 1D: at 20 °C, this parameter is voltage-dependent as previously reported [13,19,20], and increases at negative potentials, with values between 5 and 20 μM. At 30 °C, and in the same oocytes, *K*_05_ is significantly larger at all potentials, while the voltage-dependence is qualitatively maintained.

### 2.2. Apparent Affinity Changes Induced by Temperature in KAAT1

The same kind of experiments was repeated in another transporter, the *Manduca sexta* intestinal transporter KAAT1. Although clearly not a neurotransmitter transporter, this protein has a significant homology with the mammalian neutral amino acid transporter B^0^AT1, another member of the SLC6A family [21,22]. KAAT1 shows several distinct features, among which is the capability to accept various neutral amino acid as substrates and to use potassium as a driving ion [18].

In this case, threonine was used as a substrate, since this amino acid elicits a large transport current in KAAT1 when the driving ion is sodium [23].

**Figure 2:**
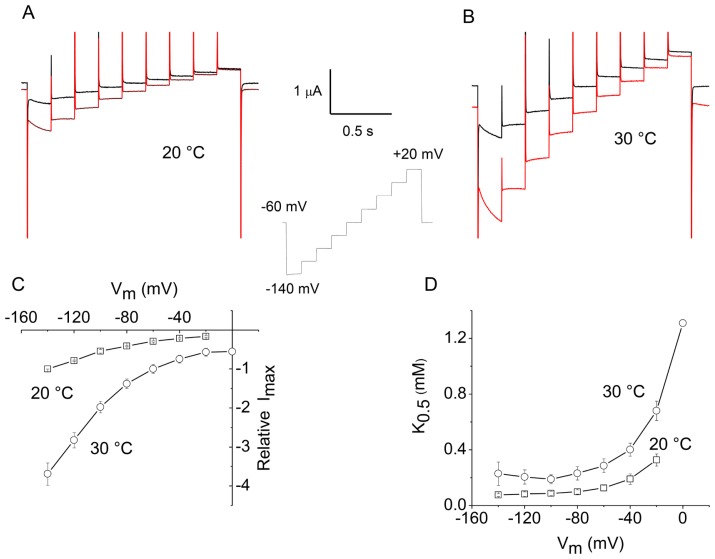
Temperature effects on the kinetic properties of KAAT1 with threonine as a substrate. Top row: currents in the absence (black) or presence (red) of 1 mM threonine at 20 °C (**A**) and 30 °C (**B**) in response to the staircase voltage protocol shown in the inset. The bottom row shows the results of the Michaelis-Menten analysis performed on dose-response curves obtained using the same experimental protocol: voltage dependence of *I*_max_ (**C**) and of *K*_05_ (**D**) at the indicated temperatures. Data are means ± SE from six oocytes (two batches). The current data were normalized to the value at −120 mV and 20 °C for each oocyte before averaging. Some *I*_max_ and *K*_05_ values at the most positive potentials are omitted because their estimate is unreliable. All data at 30 °C (except the *K*_05_ value at −140 mV) were significantly different (*p* < 0.05) from those at 20 °C (Student’s *t*-test).

The results of these experiments are shown in Figure 2. In this case, the holding potential was kept at −60 mV, and the voltage protocol covered the interval −140 to +20 mV to account for the more negative operating range of this transporter [11]. The staircase consisted of nine 20 mV steps of shorter duration compared to those used for rGAT1, because the decline of the presteady-state currents is much faster in this transporter.

Dose-response curves were obtained in this case as well, and the data analyzed with the Michaelis-Menten equation (Equation 1). As illustrated in Figure 2C, raising the temperature from 20 to 30 °C produces a considerable increase in *I*_max_, which is, however, not as large as in the case of rGAT1 (Figure 1C), but is, instead, similar to that observed in the oligopeptide transporter PepT1 [12], with *Q*_10_ values between 3 and 4. The action of temperature on *K*_05_ is shown in Figure 2D. It may be noted that the voltage-dependence of this parameter at 20 °C is different from that exhibited by rGAT1 (Figure 1D), *i.e.*, it shows an increase at more positive potentials, rather than a decrease; in fact this behavior confirms previous determinations [24], and it is very likely related to the different characteristics of the presteady-state currents in the two transporters [11,16]. Concerning the effect of temperature, a significant increase in the value of *K*_05_ is evident at 30 °C, compared to 20 °C, although, in analogy with Figure 1D, the shape of the curve is qualitatively unchanged.

The results illustrated above for rGAT1 and KAAT1 confirm the effect already observed in rabbit PepT1 [12] and suggest that a change in apparent affinity with temperature might be a feature shared by different transporters, even those belonging to diverse gene families. It must be noted, however, that some results indicating no significant effects have been also reported in other transporters [25].

### 2.3. Overall Efficiency

**Figure 3:**
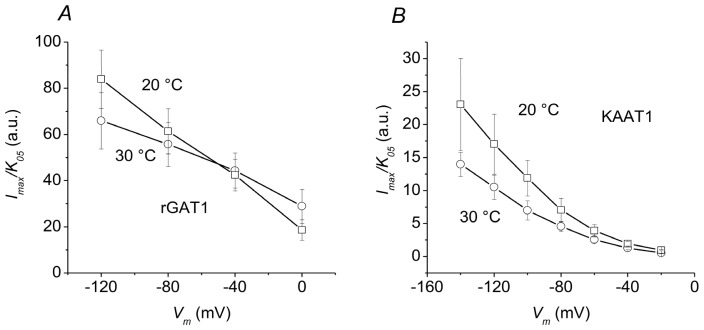
Temperature dependence of the efficiency of transport. The ratios *I*_max_/*K*_05_ have been calculated from the data in Figure 1C,D for rGAT1 and from the data in Figure 2C,D for KAAT1. The values are in arbitrary units because of the normalization of the maximal current in Figures 1 and 2. Error bars are standard errors of the mean.

The increase in *I*_ma_*_x_* and the decrease in apparent affinity reported above will counteract each other in determining the overall efficiency of the process. According to enzyme kinetics criteria [26], this parameter may be estimated as the ratio *I*_max_/*K*_05_, and it is plotted in Figure 3 for the two transporters.

In both cases, the transport efficiency increases as the membrane potential is made more negative. However, no statistically significant differences between 20 and 30 °C are observed in both transporters, especially in the physiological range of membrane potentials, around −70 mV for a neuronal presynaptic membrane, or surrounding glial cells, where rGAT1 is generally located, and at about −200 mV for the luminal side of absorptive intestinal cells, in the case of KAAT1 [27].

## 3. Experimental Section

### 3.1. Oocyte Expression

Oocytes and RNAs were prepared as previously described in detail [28]. Briefly, to prepare the mRNA, the cDNA encoding the rGAT1 and KAAT1 proteins cloned into the pAMV vector (generous gift from C. La Barca and H. Lester) were linearized with NotI; cRNAs were then synthesized *in vitro* in the presence of Cap Analog and 200 units of T7 RNA polymerase. All enzymes were supplied by Promega Italia (Milan, Italy). Adult female *Xenopus laevis* (Xenopus Express, Le Bourg, Vernassal, Haute-Loire, France) were anaesthetized in MS222 (tricaine methansulfonate, Sigma-Aldrich srl, Milan, Italy) 0.10% (*w*/*v*) solution in tap water, and portions of the ovary were removed through an incision on the abdomen. These procedures were carried out according to institutional and national ethical guidelines (permit no12/09). The oocytes were treated with collagenase Type IA, (Sigma-Aldrich srl, Milan, Italy) 1 mg/mL in calcium-free ND96, for at least 1 h at 18 °C. After 24 h at 18 °C in modified Barth’s saline solution (MBS), selected oocytes were injected with 12.5 ng of cRNA in 50 nL of water, using a manual microinjection system (Drummond Scientific Company, Broomall, PA, USA). The oocytes were then incubated at 18 °C for 3–4 days in MBS before electrophysiological studies.

### 3.2. Electrophysiology and Data Analysis

Transport activity was estimated in terms of the transmembrane current generated under voltage control, using the two-electrode voltage-clamp technique (TEVC, Oocyte Clamp OC-725B, Warner Instruments, Hamden, CT, USA). Intracellular glass microelectrodes were filled with KCl 3 M and had tip resistances between 0.5–4 MΩ. Agar bridges (3% agar in 3 M KCl) connected the bath electrodes to the experimental chamber.

The experiments were run under the WinWCP version 4.4.6 software (J. Dempster, University of Strathclyde, UK, 2012). Data were analyzed using Clampfit 10.2 (Molecular Devices, LLC, Sunnyvale, CA, USA, 2012), while figures were prepared with Origin 8.0 (OriginLab Corp., Northampton, MA, USA, 2008).

As usual, the transport-associated current was obtained by subtracting the records in the absence of substrate from those in its presence.

### 3.3. Temperature Control

Cold solutions in the reservoirs were heated to the desired temperature just before entering the recording chamber. A TC-344A in-line heater controller (Warner Instr. Corp., Hamden, CT, USA) with feedback control of the temperature was used. The actual temperature in close proximity of the oocyte was continuously monitored through a second thermistor placed in the bath. The effects of small temperature oscillations were minimized by using short-duration protocols, such as the voltage staircases mentioned in the Results and Discussion section.

### 3.4. Solutions

The oocyte culture and washing solutions had the following composition (in mM): ND96: NaCl 96, KCl 2, MgCl_2_ 1, CaCl_2_ 1.8, Hepes 5, pH 7.6; MBS: NaCl 88, KCl 1, NaHCO_3_ 2.4, Hepes 15, Ca(NO_3_)_2_ 0.30, CaCl_2_ 0.41, MgSO_4_ 0.82, sodium penicillin 10 μg/mL, streptomycin sulphate 10 μg/mL, gentamycin sulphate 100 μg/mL, nystatin 10 U/mL, pH 7.6; PBS: NaCl 138, KCl 2.7, Na_2_HPO_4_, KH_2_PO_4_, pH 7.6.

The external control solution during the electrophysiological recordings had the following composition (mM): NaCl, 98; MgCl_2_, 1; CaCl_2_, 1.8, Mes 5 mM. The final pH value (7.6) was adjusted with HCl and NaOH.

The substrates were added to this solution at the desired concentrations. For the dose-response experiments, the GABA concentrations were 1, 3, 10, 30, 100 and 300 μM; the threonine concentrations were 3, 10, 30, 100, 300 and 1000 μM.

## 4. Conclusions

All ion-coupled cotransporters tested so far to investigate the effects of temperature have shown changes in transport activity with *Q*_10_ values of about three or more [5–8,12,29]. These large effects, imply high activation energies for the rate-limiting step in the process, in the range of several tens of kiloJoules per mole (kJ/mol) and confirm, therefore, that the transport process must involve a relevant conformational change of the protein.

For mammalian transporters, the observations obtained in these studies might lead to the expectation that, at their physiological body temperature, they might be much more efficient in substrate translocation. However, the overall transport efficiency will obviously depend on another property of the transporter: its apparent affinity for the substrate.

Relatively little attention has been paid to this second aspect, and published reports are scarce. Some results implying a decrease in apparent affinity at low temperatures can be found in an earlier paper on the noradrenaline transporter [30], and subsequently, similar results were reported in the Drosophila serotonin transporter [8]. On the contrary, no temperature-induced changes in apparent affinity were observed in the human form of GAT1 [25]. In a recent study on the intestinal PepT1 transporter from our laboratory [12], we confirmed the earlier observations, and we show here that two other cotransporters, the rat neuronal GABA transporter GAT1 implied in many important physiopathological issues, and the invertebrate intestinal amino acid transporter KAAT1, also exhibit this same feature.

The explanation for this behavior may be directly found in the transport mechanism, which for the transporters under consideration appears very similar [13,24,31,32]. In these transporters, in fact, the initial steps of the cycle, involving the interaction with the driving ion(s) and the following intramembrane charge movement, precede the binding of the organic substrate. Furthermore, these steps are rate-limiting of the entire transport cycle [31–33]. Consequently, the lifetime of the conformational state in which substrate binding can occur will be shortened when the turnover rate is increased (e.g., in the present case, by a higher temperature). A shorter life time of this state implies that a higher concentration of substrate will be needed to sustain the high turnover rate, leading therefore to a decreased apparent affinity.

The membrane composition of the *Xenopus* oocyte is unlikely to be the same of a mammalian or of a *Manduca* cell, and therefore, the present results cannot be directly extrapolated to the respective native conditions. We show here that the increased *I*_max_ at higher temperature is counterbalanced by a lower apparent affinity, and that in both rGAT1 and KAAT1, the two effects approximately compensate for each other, so that the efficiency, estimated as the ratio *I*_max_/*K*_05_, appears substantially temperature-independent. However, in the case of the GABA transporter, the lower apparent affinity at higher temperature is likely to produce consequences on the basal extracellular levels of the neurotransmitter, which are known to be very important in neurological pathophysiology [34,35].
